# 2485 Advancing research professionals through competency assessments

**DOI:** 10.1017/cts.2018.195

**Published:** 2018-11-21

**Authors:** Rebecca N. Brouwer, Denise Snyder, Deborah Hannah, Christine Deeter

**Affiliations:** Duke University Medical Center

## Abstract

OBJECTIVES/SPECIFIC AIMS: Describe the framework for tier advancement of research professionals. Describe the various forms of assessments of competencies. How competencies are used to provide transparency into professional development opportunities. Discuss the results of the first tier advancement opportunity for research staff. METHODS/STUDY POPULATION: These processes were developed at Duke, an academic medical center with over 2000 active clinical research protocols and 300 new clinical trials per year. Roughly 500 employees are categorized into tiered classifications, allowing them opportunities for advancement through competency testing. Approximately 10% opted for tier testing, and their results will be shared. RESULTS/ANTICIPATED RESULTS: Competency assessments were developed for all 42 of Duke’s research professional competencies, some using 2 modalities of testing. Almost 12% of the research professionals classified in tiered positions opted to attempt the tier advancement process. Of those, 37 completed, and the vast majority reached their desired tier. Results by competency will be provided. DISCUSSION/SIGNIFICANCE OF IMPACT: The use of objectively assessed competencies is an important step in the development of a workforce. By (1) maintaining alignment with industry standards for competencies, (2) holding staff to a high bar, and (3) offering a consistent approach to career growth, Duke is working to develop and maintain a workforce that supports high quality research.

